# Computed Tomography Radiomic Nomogram for Preoperative Prediction of Extrathyroidal Extension in Papillary Thyroid Carcinoma

**DOI:** 10.3389/fonc.2019.00829

**Published:** 2019-09-04

**Authors:** Bin Chen, Lianzhen Zhong, Di Dong, Jianjun Zheng, Mengjie Fang, Chunyao Yu, Qi Dai, Liwen Zhang, Jie Tian, Wei Lu, Yinhua Jin

**Affiliations:** ^1^Department of Medical Imaging, Hwa Mei Hospital, University of Chinese Academy of Sciences, Ningbo, China; ^2^CAS Key Laboratory of Molecular Imaging, Institute of Automation, Chinese Academy of Sciences, Beijing, China; ^3^School of Artificial Intelligence, University of Chinese Academy of Sciences, Beijing, China; ^4^Beijing Advanced Innovation Center for Big Data-Based Precision Medicine, School of Medicine, Beihang University, Beijing, China

**Keywords:** thyroid cancer, computed tomography, radiomics, tumor staging, nomograms

## Abstract

**Objectives:** Determining the presence of extrathyroidal extension (ETE) is important for patients with papillary thyroid carcinoma (PTC) in selecting the proper surgical approaches. This study aimed to explore a radiomic model for preoperative prediction of ETE in patients with PTC.

**Methods:** The study included 624 PTC patients (without ETE, *n* = 448; with minimal ETE, *n* = 52; with gross ETE, *n* = 124) whom were divided randomly into training (*n* = 437) and validation (*n* = 187) cohorts; all data were gathered between January 2016 and November 2017. Radiomic features were extracted from computed tomography (CT) images of PTCs. Key radiomic features were identified and incorporated into a radiomic signature. Combining the radiomic signature with clinical risk factors, a radiomic nomogram was constructed using multivariable logistic regression. Delong test was used to compare different receiver operating characteristic curves.

**Results:** Five key radiomic features were incorporated into the radiomic signature, which were significantly associated with ETE (*p* < 0.001 for both cohorts) and slightly better than clinical model integrating significant clinical risk factors in the training cohort (area under the receiver operating characteristic curve (AUC), 0.791 vs. 0.778; F_1_ score, 0.729 vs. 0.714) and validation cohort (AUC, 0.772 vs. 0.756; F_1_ score, 0.710 vs. 0.692). The radiomic nomogram significantly improved predictive value in the training cohort (AUC, 0.837, *p* < 0.001; F_1_ score, 0.766) and validation cohort (AUC, 0.812, *p* = 0.024; F_1_ score, 0.732).

**Conclusions:** The radiomic nomogram significantly improved the preoperative prediction of ETE in PTC patients. It indicated that radiomics could be a valuable method in PTC research.

## Key points

* Conventional imaging diagnostic methods are limited in the assessment of ETE.
* Radiomic signature is complementary to clinical data and conventional CT in assessing ETE.
* Radiomic nomogram can improve the preoperative prediction of ETE in patients with PTC.

## Introduction

Thyroid carcinoma is one of the most common endocrine system malignancies, with a rapidly increasing incidence worldwide in recent years (1–3). Papillary thyroid carcinoma (PTC) is the most common type, accounting for nearly 90% of all thyroid carcinomas (4). PTC usually has an excellent prognosis and, to reduce postoperative complications, many researchers recommend ipsilateral lobectomy rather than total thyroidectomy for low-risk patients with PTC (1, 5, 6). Compared with total thyroidectomy, unilateral lobectomy has no significant differences in terms of distant metastasis and cancer-specific mortality rates (7). Extrathyroidal extension (ETE) is one of the criteria in choosing appropriate surgical management (1). ETE is divided into minimal and gross ETE based on the degree of invasion into the surrounding structures. According to American Joint Committee on Cancer (AJCC) TNM Staging for Thyroid–Differentiated and Anaplastic Carcinoma (8th Edition, 2017), minimal ETE refers to the extension of the primary tumor to the peri-thyroid soft tissues, while gross ETE means that the primary tumor invades the surrounding structures including the strap muscles, trachea, larynx, vasculature, esophagus, and recurrent laryngeal nerve (RLN). The National Comprehensive Cancer Network (NCCN) Guidelines for Thyroid Carcinoma (Version 3, 2018) recommend total thyroidectomy as the optimal primary treatment for PTC patients with gross ETE. Moreover, ETE is an independent risk factor associated with increased risk for morbidity and mortality (6). The 15-year survival rate of PTC patients with ETE is significantly lower than that of patients without ETE (6, 8). Therefore, the diagnosis of ETE is essential for the treatment decision of PTC.

Surgical histopathological examination is the gold standard for diagnosing ETE. Although fine-needle aspiration (FNA) can adequately evaluate thyroid nodules preoperatively (9), it provides little information about ETE (10). Ultrasonography (US) is the preferred imaging modality for the preoperative detection and diagnosis of PTC; however, it has limitations in assessing ETE (11, 12). Computed tomography (CT) has advantages over US for evaluating tumor extension to adjacent structures. A previous study reported that contrast-enhanced CT (CE-CT) had excellent specificity but low sensitivity in assessing ETE (13). Magnetic resonance imaging (MRI) can provide high soft tissue resolution without radiation or delaying radioiodine therapy. However, MRI is more expensive and time-consuming than US and CT, and it does not demonstrate better performance in assessing ETE compared with US (14).

Radiomics is an emerging and burgeoning subject in medical research, especially in oncology. It can provide an enormous amount of high-dimensional and quantitative imaging features that are associated with tumor gene expression, invasiveness, and prognosis (15–18). Radiomics has been applied to the preoperative prediction of lymph node (LN) metastasis (19), evaluation of occult peritoneal metastasis (20), and assessment of prognosis (21). Thus, radiomics can potentially help predict ETE based on pre-treatment CT scans. However, to our knowledge, radiomic research investigating ETE in PTC patients is currently limited.

Therefore, this study aimed to construct and validate a CT-based radiomic model, which combines radiomic signature with clinical risk factors, for the individualized preoperative prediction of ETE in patients with PTC to help clinicians choose the optimal primary treatment strategy.

## Materials and Methods

### Patient Data

The studies involving human participants were reviewed and approved by the ethics committee of Hwa Mei Hospital, University of Chinese Academy of Sciences. Written informed consent for participation was not required for this study in accordance with the national legislation and the institutional requirements. This study included 624 patients [123 men and 501 women; mean (±SD) age, 44.75 ± 13.16 years; range, 19–76 years] who underwent thyroid surgery between January 2016 and November 2017 (Figure S1). The inclusion criteria were as follows: (1) the primary tumor was confirmed to be PTC by histopathological examination; (2) CE-CT was performed before surgery; and (3) ipsilateral lobectomy or total thyroidectomy were undergone. The exclusion criteria were as follows: (1) primary tumors not clearly visible on CT images due to artifacts; (2) primary tumor with a maximum primary tumor diameter < 5 mm; and (3) tumors that could not be distinguished from nodular goiter, chronic lymphocytic thyroiditis or other thyroid diseases on CT imaging of the surrounding thyroid tissues. According to the histopathological diagnosis, patients were divided into those without ETE (*n* = 448), with minimal ETE (*n* = 52), and with gross ETE (*n* = 124). Due to the small positive sample size, patients with minimal ETE and gross ETE were categorized under the same group for ETE to enable binary classification. CT data and clinical information from the patients were collected for this study. Details of CT acquisition and the retrieval procedure are presented in Appendix A1 in Supplementary Material.

### Radiologists' Prediction of ETE

CT assessment of ETE (i.e., radiologists' prediction of ETE) was performed by two radiologists, each with > 10 years' experience. Any disagreements were resolved by consensus or the consultation with a third radiologist with > 20 years' experience. Based on previous studies (13, 22–24), ETE was suspected on CT images when at least one of the following criteria were present: (1) the percentage of the primary tumor perimeter in contact with the thyroid capsule was > 25%; (2) the primary tumor was in contact with ≥ 180° of the tracheal, esophageal, or vascular [common carotid artery, (CCA); internal jugular vein, (IJV)] circumference; (3) the loss of normal tracheal, esophageal, or vascular (CCA, IJV) structures (wall and lumen) at the level of the primary tumor; or (4) invasion of the RLN Appendix A2 in Supplementary Material.

### Tumor Segmentation

Segmentations were manually performed on each slice on both the reconstructed non-enhanced and venous CE-CT images by a radiologist [observer 1, (W.L.)] with > 6 years' experience using ITK-SNAP software [open source software (www.itksnap.org)]. The region of interest (ROI) covered the entire tumor and the adjacent tissue within 1 mm from the tumor boundary (Figure S2). To evaluate the reproducibility of features among different segmentations, 30 cases were selected at random and their images were re-segmented by observer 1 two weeks after the initial segmentation. Another radiologist [observer 2, (W.S.)] also performed segmentation on the 30 cases. The class correlation coefficient (CCC) was used to measure the intra- and inter-observer agreement of the extracted features, and a CCC > 0.7 indicated excellent reliability (25).

### Radiomic Feature Extraction

For each individual CT scan, algorithms defined in studies by Aerts et al. (26) and Lambin et al. (27) were programmed to automatically extract radiomic features (Appendix A3 in Supplementary Material) from the manually segmented tumor regions. All feature extraction methods were performed using MATLAB 2017b (MathWorks, Natick, MA, USA). To extract robust features, bilinear interpolation was used to normalize the in-plane voxel size of CT images to 0.5 mm before feature extraction.

### Feature Selection and Radiomic Signature Building

All data were randomly divided into training and validation sets at a ratio of 7:3. All predictive models were developed on the training cohort. Different combinations of feature selection and machine learning classifiers were compared for preoperative prediction of ETE in PTC. Least absolute shrinkage and selection operator (LASSO), principal component analysis, and minimum redundancy maximum relevance were used to screen out discriminative features. Logistic regression, random forest, and support vector machine were adopted to build predictive models. A description of the featured selection methods and machine learning classifiers are elaborated on in Appendix A4 in Supplementary Material. We selected the best combination using 10-fold cross-validation, with area under of receiver operating characteristic (ROC) curve (AUC) as a performance indicator. The prediction for the best classifier, which was termed the “radiomic signature,” was binarized to gain sensitivity and specificity. The cut-off was selected in the training cohort and used in the validation cohort.

### Predictive Validation of the Radiomic Signature

The potential association of our radiomic signature with ETE was first evaluated in the training cohort using the chi-square test before being applied to the validation cohort. The performance of the radiomic signature was compared with the radiologists' prediction of ETE.

### Construction and Performance of the Radiomic Nomogram

Monofactor analysis was used to explore the association between the clinical risk factors and ETE of PTCs, and then a clinical model was constructed in multivariable logistic regression analysis. To investigate the incremental value of the radiomic signature for prediction of ETE in PTCs, the radiomic model was built by combining the radiomic signature with clinical predictors. This was then converted into a radiomic nomogram for providing clinicians with a visual tool to predict the individual probability of ETE in PTC patients. The differences between the ROC curves of the radiomic and clinical models were compared using the DeLong test (28, 29).

### Validation of the Radiomic Nomogram

The predictive performances of the clinical and radiomic nomograms were evaluated in the training cohort and then tested in the validation cohorts. Calibration curves from the radiomic nomogram were obtained from the training and validation cohorts and then assessed using the Hosmer-Lemeshow test (30). Stratified analyses were performed to test the predictive ability of the radiomic nomogram in various subgroups of the entire data set.

### Clinical Practice

To estimate the incremental utility of the radiomic signature, the decision curve of the different models was plotted for the entire dataset. The decision curve informs a patient or doctor which of several models (if any) is the optimal using a threshold probability (31).

### Statistical Analysis

The Delong test was used to compare the different ROC curves and F_1_ score to assess classification performance. F_1_ score, defined as the harmonic mean of the sensitivity and specificity of a classification model, is an indicator of the accuracy of a binary model. All AUCs are reported with corresponding 95% confidence interval (CI). Continuous variables were tested using the *t*-test or the Wilcoxon rank sum test while categorical variables were analyzed using the Pearson's χ^2^ test or the Fisher's exact test. Statistical analyses were performed using the R software (http://www.R-project.org). The R packages (Appendix A5 in Supplementary Material) were used as radiomic features extraction methodology. A two-sided *p* < 0.05 was considered to be statistically significant.

## Results

### Clinical Characteristics of the Patients

The training cohort comprised 437 PTCs with a positive ETE rate of 27.9%, while the validation cohort comprised 187 PTCs with a positive ETE rate of 28.9%. No significant differences were found between both cohorts in terms of any relevant clinical risk factors (Table S1). The associations between the presence of ETE and the clinical risk factors in training cohort are summarized in Table 1. Body mass index (BMI) and LN metastasis status did not demonstrate significant association with ETE. The radiologists' prediction of ETE had a F_1_ score of 0.619 but a poor sensitivity (0.477) in the entire data set.

**Table 1:** Associations between extrathyroidal extension and clinical risk predictors in training cohort.

**Characteristics**	**ETE (-) (N = 315)**	**ETE (+) (N = 122)**	***p***
Age, mean ± SD, years	44.31 ± 12.76	46.91 ± 13.31	0.060
< 55, *N* (%)	245 (77.78)	87 (71.31)	0.195
≥ 55, *N* (%)	70 (22.22)	35 (28.69)	
Sex, *N* (%)			
Male	66 (20.95)	14 (11.48)	0.031
Female	249 (79.05)	108 (88.52)	
Primary site (Location), *N* (%)			
Right/Left lobe	307 (97.46)	112 (91.80)	0.016
Isthmus	8(2.54)	10 (8.20)	
Primary site (Position, A-P), *N* (%)			
Ventral	112 (35.56)	67 (54.92)	0.001
Medium	12 (3.81)	3 (2.46)	
Dorsal	191 (60.63)	52 (42.62)	
Diameter, mean ± SD, mm	10.88 ± 5.93	15.22 ± 7.49	< 0.001
Calcification, *N* (%)			
Negative	209 (66.35)	61 (50.00)	0.002
Positive	106 (33.65)	61 (50.00)	
BMI, mean ± SD, kg/m^2^	22.78 ± 3.11	23.08 ± 3.38	0.365
< 25, *N* (%)	238 (75.56)	93 (76.23)	0.982
≥ 25, *N* (%)	77 (24.44)	29 (23.77)	
Radiologists' prediction of ETE, *N* (%)			
Negative	281 (89.21)	61 (50.00)	< 0.001
Positive	34 (10.79)	61 (50.00)	
LN metastasis, *N* (%)			
Negative	144 (45.71)	52 (42.62)	0.634
Positive	171 (54.29)	70 (57.38)	

*ETE, extrathyroidal extension; BMI, body mass index; SD, Standard Deviation; LN, lymph node*.

### Radiomic Feature Extraction/Selection

A total of 546 features were extracted for each patient in both the training and validation cohorts (273 features from non-enhanced images and the remainder from venous contrast-enhanced images). A total of 480 (88%) radiomic features with CCCs > 0.7 (Figure S3) were reserved for subsequent analysis. The combination of LASSO and logistic regression identified five key features and showed the best result (AUC, 0.781) among all schemes (Table S2).

### Radiomic Signature Construction

The radiomic signature generated by the multivariable logistic regression model was based on the five reserved key features. The weight of each feature in the radiomic signature was calculated based on its respective coefficient (Appendix A6 in Supplementary Material).

### Predictive Validation of the Radiomic Signature

The association between the radiomic signature and ETE was found to be significant in the training cohort (*p* < 0.001), which was subsequently confirmed in the validation cohort (*p* < 0.001). The radiomic signature demonstrated good predictive performance in the training (AUC, 0.791 [95% CI, 0.745–0.837]; F_1_ score, 0.729) and validation (AUC, 0.772 [95% CI, 0.700–0.844]; F_1_ score, 0.710) cohorts, which was better than the radiologists' prediction of ETE (F_1_ score, 0.619 for the entire dataset). The clinical model identified age, radiologists' prediction of ETE, and tumor position as independent variables, with a slightly lower discrimination than the radiomic signature (AUC, 0.778 [95% CI, 0.726–0.829], F_1_ score, 0.714 in the training cohort; AUC, 0.756 [95% CI, 0.679–0.834], F_1_ score, 0.692 in the validation cohort. Figure S4).

### Construction and Performance of the Radiomic Nomogram

**Figure 1:**
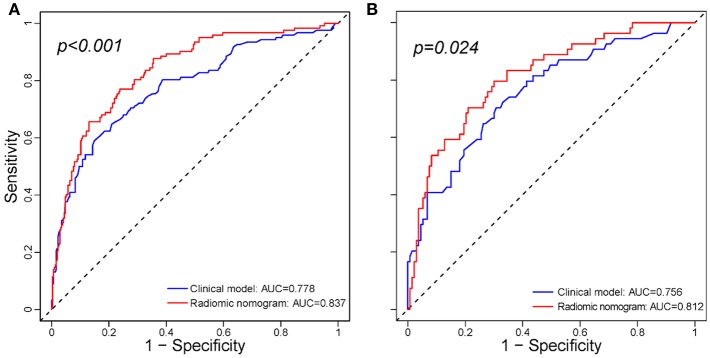
Comparison between the clinical model and radiomic nomogram. **(A)** Comparison ROC curves between the clinical model and radiomic nomogram in the training cohort. **(B)** Comparison ROC curves between the clinical model and radiomic nomogram in the validation cohort. ROC, receiver operating characteristic; AUC, area under receiver operating characteristic curve.

**Figure 2:**
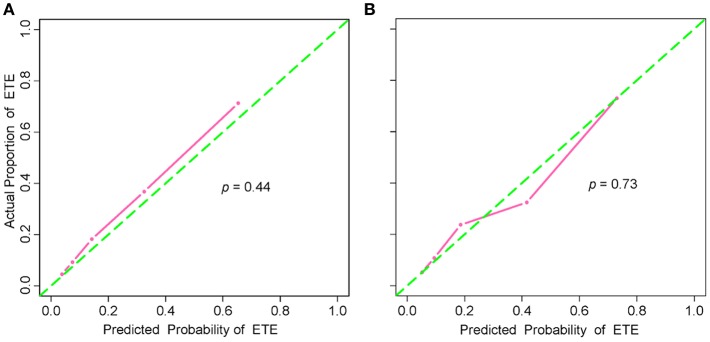
Calibration curves of the radiomic nomogram in the training and validation cohorts. **(A)** Calibration curve of the radiomic nomogram in the training cohort. The Hosmer-Lemeshow test yielded a non-significant statistic (*p* = 0.44). **(B)** Calibration curve of the radiomic nomogram in the validation cohort. The Hosmer-Lemeshow test also yielded a non-significant statistic (*p* = 0.73). Calibration curves describe the model's calibration in term of agreement between the predicted probability of ETE and observed positive proportion of ETE. The green dashed line represents perfect performance, while the pink solid line presents the actual performance of the radiomic nomogram. ETE, extrathyroidal extension.

**Figure 3:**
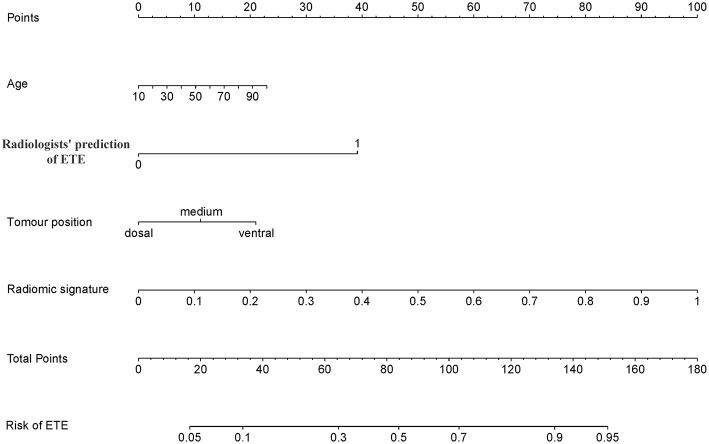
Radiomic nomogram. The radiomic nomogram incorporated age, radiologists' prediction of ETE, tumor position and the radiomic signature. For example, the primary tumor of a 50-year-old PTC patient was found on the ventral side of the thyroid and was determined to have ETE by CT; its radiomic signature score was 80, the total number of points of this tumor was 150 (10 + 40 + 20 + 80), and the risk rate of ETE was determined to be 95%. ETE, extrathyroidal extension; PTC, papillary thyroid carcinoma.

The addition of the radiomic signature to the clinical model (i.e., radiomic nomogram) significantly improved the prediction of ETE in the training cohort, and was better than the clinical model alone (AUC, 0.837 [95% CI, 0.795–0.879] vs. 0.778 [95% CI, 0.726–0.829]; F_1_ score, 0.766 vs. 0.714). A significant difference in ROC curves from these two models was found in the training cohort (*p* < 0.001, Figure 1A). The calibration curve for the probability of ETE showed good agreement between the nomogram-predicted probability of ETE and the actual ETE observed (*p* = 0.44) (Figure 2A). Outcome measurements of all models are presented in Table S3 and the radiomic nomogram is illustrated in Figure 3.

### Validation of the Radiomic Nomogram

In the validation cohort, the radiomic nomogram also demonstrated a better performance than the clinical model (AUC, 0.812 [95% CI, 0.744–0.879] vs. 0.756 [95% CI, 0.679-0.834], *p* = 0.024, Figure 1B; F_1_ score, 0.732 vs. 0.692). Regarding the calibration curves, good agreement (*p* = 0.73) was found between the nomogram-predicted probability of ETE and the actual observed ETE (Figure 2B).

**Figure 4:**
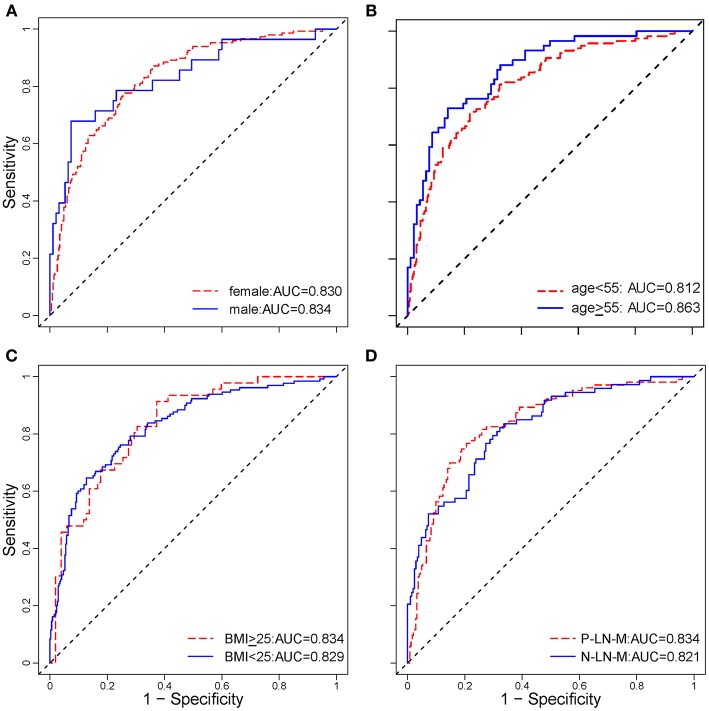
Stratified analysis of the radiomic nomogram in different subgroups. Stratified analysis for ETE in patients with PTC according to sex **(A)**, age **(B)**, BMI **(C)**, and LN metastasis status **(D)**. The age cut-off was based on the National Comprehensive Cancer Network (NCCN) Guidelines for thyroid carcinoma (Version 3. 2018). These analyses demonstrated that the radiomic nomogram had good and similar discriminations for predicting ETE status in patients with PTC in different subgroups. ETE, extrathyroidal extension; PTC, papillary thyroid carcinoma; AUC, area under receiver operating characteristic curve; BMI, body mass index; P-LN-M, positive lymph node metastasis; N-LN-M, negative lymph node metastasis.

Stratified analysis revealed that the radiomic nomogram demonstrated good and stable prediction for ETE in the different subgroups (Figure 4), which confirmed the robustness of the model. Additionally, 85.4% patients of the 52 patients with minimal ETE, and 80.6% of the 124 patients with gross ETE were successfully identified in the entire dataset.

### Clinical Practice

**Figure 5:**
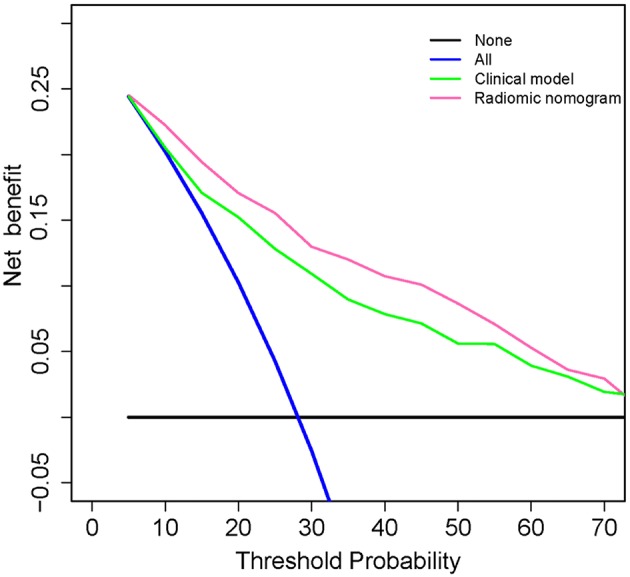
Decision curve analysis for clinical and radiomic nomograms. The black solid line represents the assumption that all patients with PTC do not have ETE. The blue solid line represents the assumption that all patients with PTC have ETE. The green solid line represents the assumption that patients with PTC will be judged positive if the positive probability obtained from clinical nomogram is higher than the threshold probability. The red solid line represents the assumption that patients with PTC will be judged positive if the positive probability obtained from the radiomic nomogram is higher than the threshold probability. The decision curves showed that when the threshold probability is in a reasonable range, the radiomic nomogram provided the greatest net benefit when compared with the treat-all-patients approach, treat-none approach, and the clinical nomogram for predicting ETE status of patients with PTC. ETE, extrathyroidal extension; PTC, papillary thyroid carcinoma.

It has been suggested that net benefits should be compared when the threshold probability is between 2 and 50 (32). As shown in Figure 5, using the radiomic nomogram to predict ETE produced the greatest benefit in this range, which demonstrated the high value of the radiomic nomogram.

## Discussion

PTC patients with ETE have an increased risk for morbidity and mortality and require total thyroidectomy. Thus, accurate preoperative prediction of ETE can help surgeons to determine an appropriate surgical management strategy to reduce the risk for re-operation. In this study, radiologists' prediction of ETE had a good specificity but a low sensitivity. A total of 176 patients had pathologically confirmed ETE while only 84 had suspected ETE according to CT findings. Thus, increasing the sensitivity is crucial to improving the accuracy of ETE diagnosis.

In this study, we developed a CT-based radiomic method for preoperative assessment of ETE. Small (< 5 mm) and unclear tumors were excluded to avoid inaccurate segmentation. The radiomic signature achieved a better classification performance than radiologists' prediction of ETE (F_1_ score, 0.710 vs. 0.619) and had a slightly higher predictive value than that of the clinical model (AUC, 0.772 vs. 0.756) in the validation cohort, which demonstrated the impressive prediction ability of radiomic signature. Moreover, adding the radiomic signature to the clinical model led to significant improvements in both the training (AUC, 0.778 to 0.837, *p* < 0.001) and validation (AUC, 0.756 to 0.812, *p* = 0.024) cohorts. Furthermore, the radiomic nomogram demonstrated good agreement in calibration and had the highest net benefit within a reasonable range of threshold probabilities. These findings demonstrated the impressive predictive ability of the radiomic signature.

The criterion of minimal ETE for T-staging has been removed from AJCC/TNM system version 8. One reason is that minimal ETE is not an independent risk factor associated with prognosis for patients with PTC (33–35). However, the change has sparked widespread controversy, and some specialists put proposed a different perspective in which minimal ETE would increase the risk for recurrence (36, 37). Therefore, both minimal and gross ETE were regarded as ETE-positive for analysis in this study.

The criteria for radiologists' prediction of ETE were determined based on those reported in previous studies (13, 22), and our results also demonstrated that CT had good specificity (87.9%) but low sensitivity (47.7%) in predicting ETE. Previous studies have shown that older patients (> 55 years of age) and males with PTC exhibited decreased survival rates (38). This suggested that age and sex were related to degree of malignancy and prognosis of PTC, and may also be associated with ETE. Additionally, tumors on the ventral side of the thyroid or those with calcifications tended to demonstrate greater association with ETE, which may provide complementary information for precise assessment. A previous study reported that patients with papillary thyroid microcarcinoma and a BMI ≥ 25 kg/m^2^ exhibited a higher prevalence of ETE than those with a BMI < 25 kg/m^2^ (39). However, in our study, there was no significant difference between PTC patients with or without ETE (*p* = 0.432) in terms of BMI. Our radiomic signature demonstrated better performance than radiologists' prediction of ETE and the clinical model. It suggested that the selected features contain more information that was significantly associated with ETE but not considered to be conventional risk factors. For example, compactness indicates tumor shape, homogeneity and sum-variance from the gray level co-occurrence matrix indicate unevenness of the tumor density and enhancement, and all of these features are invisible to the human eye but associated with tumor heterogeneity.

US is the most widely used imaging modality for PTC staging. It can clearly depict the degree of contact between the tumor and the adjacent thyroid capsule and the disruption of the capsule. In a previous study, Gweon et al. used sonography for preoperative assessment of ETE in 79 cases of PTC, with an accuracy of 60.8% (2D) and 66.2% (3D) (12). Seo et al. estimated the diagnostic accuracy of CT for detecting gross ETE in 84 patients with PTC (13). The results demonstrated excellent specificity but limited sensitivity. In a study involving 377 PTC patients, Lee et al. reported that US findings of capsule disruption had a better AUC (0.674 vs. 0.638) in predicting ETE than that of CT findings for > 50% contact between the tumor and capsule (22), while the combination of US and CT provided the best diagnostic accuracy (sensitivity, 92.9%; specificity, 70.4%; AUC 0.744). Although we did not compare the prediction performance between US and the CT-based radiomic nomogram in this study, the AUC (0.812 vs. 0.744) of our radiomic nomogram was significantly higher than the data reported by Lee et al.

There were, however, several limitations to our investigation. First, this was a retrospective study, the clinical procedures were not strict, and some patients had incomplete laboratory examination data, such as arterial phase images that were not used for analysis due to a lack of storage of reconstructed data. Second, 193 patients did not undergo any follow-up therapy or surveillance after surgery. Due to the excellent prognosis of PTC, recurrence or new emergence of metastasis were found in only two patients. Thus, survival and prognostic analyses were not performed in this study. Third, the exclusion criteria may have caused a selection bias when small or indistinct lesions were rejected, most of which were without ETE; this may have affected model training. Fourth, because the study cohort was from a single institution, the dataset does not necessarily represent the entire PTC population. Fifth, our model was based on CT images, for which the use of iodinated contrast would delay radioiodine therapy.

In conclusion, our radiomic nomogram may improve the preoperative prediction of ETE in patients with PTC. The radiomic signature is complementary to clinical data and conventional CT.

## Data Availability

The datasets generated for this study are available on request to the corresponding author.

## Author Contributions

BC, LZho, DD, JZ, YJ, WL, and JT contributed conception and design of the study. BC and WL organized the database. LZho and DD performed the statistical analysis. BC and LZho wrote the first draft of the manuscript. JZ, MF, CY, QD, and LZha checked results of the statistical analysis. All authors contributed to manuscript revision, read and approved the submitted version.

### Conflict of Interest Statement

The authors declare that the research was conducted in the absence of any commercial or financial relationships that could be construed as a potential conflict of interest.

## Abbreviations

AUC: Area under the curve
CT: Computed tomography
ETE: Extrathyroidal extension
FNA: Fine-needle aspiration
LASSO: Least absolute shrinkage and selection operator
LN: Lymph node
MRI: Magnetic resonance imaging
NPV: Negative predictive value
PET: Positron emission tomography
PPV: Positive predictive value
PTC: Papillary thyroid carcinoma
RAI: Radioactive iodine
ROC: Receiver operating characteristic curve
SPECT: Single-photon emission computed tomography
US: Ultrasonography.
