# Supplementary material for: Computed Tomography Radiomic Nomogram for Preoperative Prediction of Extrathyroidal Extension in Papillary Thyroid Carcinoma
Source: Front Oncol. 2019 Sep 4;9:829. doi: 10.3389/fonc.2019.00829 (PMC6736997; doi:10.3389/fonc.2019.00829)
Supplement: Supplementary file 1 [file Table_1.DOCX]

**Appendix for “Computed Tomography Radiomic Nomogram for Preoperative Prediction of Extrathyroidal Extension in Papillary Thyroid Carcinoma”**

# Appendix A1. The CT data acquisition and retrieval procedure

All patients underwent CE-CT before surgery on a 16-slice spiral CT scanner (Sensation 16, SIEMENS, Erlangen, Germany) or a 64-slice spiral CT scanner (SOMATOM Definition, SIEMENS, Erlangen, Germany). The CT scan parameters were as follows: 120 kV; 150–200 mAs; pitch, 1; rotation time, 1.0 s; detector collimation, 16 × 0.75 mm or 64 × 0.6 mm; field of view, 500 × 500 mm; matrix, 512 × 512; and slice thickness, 5.0 mm. After a routine non-enhanced scan, CE-CTs were performed after a delay of 20 s (arterial phase) and 35 s (venous phase) following the intravenous administration of 85 mL of an iodinated nonionic contrast agent (iohexol injection, 350 mg I/mL, Yangtze River, Taizhou, China) at a rate of 2.5–3.0 mL/s with a high-pressure syringe (SCT-210; MEDRAD). The image reconstruction was performed on an image post-processing workstation (Syngo CT 2012B, VA44A, Siemen Medical Solution). These CT images were retrieved from the picture archiving and communication system (PACS) (eWorld, China). All images were stored, read, and processed in the DICOM data format.

# Appendix A2. The criteria of invasion of RLN

Invasion of RLN was considered when at least two of the following criteria were present: (1) complete disappearance of the fat space in the tracheoesophageal groove; (2) more than 25% of the circumference of the primary tumor in contact with the posterior capsule of thyroid; (3) clinical suggestion of ipsilateral vocal cord palsy or CT findings such as a paramedian cord, anteromedial deviation of the arytenoid cartilage, an enlarged pyriform sinus, or an enlarged laryngeal ventricle. It is worth noting that the second criterion overlapped that for detection of ETE. It means that we can diagnose ETE when the second criterion present or the first and the third criterion present simultaneously.

# Appendix A3. Radiomic features extraction methodology

In the current study, 576 candidate radiomic features were extracted, of which 273 features (with .x as a suffix) were from non-contrast CT images and the remaining (with .y as a suffix) were from venous contrast-enhanced CT images. All features were divided into four types: first-order statistics features, shape-and-size-based features, statistics-based textural features, and features after wavelet filtering. The undecimated wavelet transform was applied to decompose the original image into 4 decompositions. L and H denote a low-pass and high-pass functions respectively and X denotes the decomposing image. The wavelet decompositions of X to be labeled as X_LL_, X_LH_, X_HL_ and X_HH_, from which the features were prefixed with X1, X2, X3 and X4 respectively. Wavelet features were made up of the above first order and textural features on each decomposition. Note that the extraction of the 3D textural features requires the same voxel size in the three directions (x, y, and z direction). However, the image thickness in the z direction (3.11 ± 0.816 mm in non-enhanced images and 3.02 ± 0.750 mm in venous contrast-enhanced images) was much larger than the in-plane voxel size in the x and y direction (0.5 mm after bilinear interpolation). Therefore, the H vs L wavelets were applied in 2 directions with 4 decompositions and texture matrices were determined considering the transverse 8-connected voxels [1].

[1]. Zwanenburg A LS, Vallières M: Image biomarker standardisation initiative. arXiv preprint arXiv 2016, 1612.07003.

# Appendix A4. Comparison of different combinations of feature selection methods and machine learning classifiers

Before feature selection, we excluded poorly reproducible (n = 96) and redundant (n = 365) features. Firstly, 480 (88%) radiomic features with class correlation coefficients > 0.7 (**Appendix Figure S2**) were reserved. Secondly, the above selected features were ranked according to their significance of association with ETE; 365 features with correlation coefficients (with the former ranked features) > 0.8 were excluded. Finally, 115 features were used to perform feature selection and model construction. All radiomic features were standardized by the z-score method. Tuning parameter ($\lambda$) in the Least absolute shrinkage and selection operator (LASSO) was chosen using 10-fold cross-validation via minimum criteria. Parallel analysis was used to determine the number of principal components. Backward step-wise selection in logistic regression model was used to select variables whose coefficients reached statistical significance. Free parameters in random forest and support vector machine with radial basis function were chosen using 10-fold cross-validation, with area under of receiver operating characteristic curve (AUC) as a performance indicator. After free parameters and key features are determined, models were evaluated in the training cohort by 10-fold cross-validation. LASSO screened out 11 features, Principal Component Analysis 13 principal components and Minimum Redundancy Maximum Relevance 15 features. The combination of LASSO and logistic regression showed the best result (AUC, 0.781) among all combinations (seen in **Table S3**), which identified 5 key features.

# Appendix A5. R packages for data analysis

Intra/inter-class correlation coefficients (ICCs) between features and Principal Component Analysis were calculated by “psych” package. LASSO was performed with the “glmnet” package and mRMR with the “mRMRe” package. Random Forest was conducted by the “randomForest” package and Support Vector Machine by the “e1071” package. Multivariate binary logistic regression, nomograms and calibration plots were done with the “rms” package. ROC curve was drawn by the “ROCR” package while the test of calibration curve was conducted by the “HLtest.r” package. Decision curve analysis was performed with the function of “dca.r”.

# Appendix A6. Radiomic signature calculation formula

Through feature selection, eight radiomic features were decided and incorporated into a radiomic score by multivariable logistic regression. The radiomic signature could be obtained using:

$$radiomic signature=sigmoid(-1.404+0.795*X2.R1.y-$$

$$0.348*R2.y-0.477*X2.R3.x-$$

$$0.503*X3.R4.y-0.378*R5.x)$$

Where $\mathrm{sigmoid}\left( x \right)=\frac{1}{1+e^{-x}}$, R1 come from first-order statistics features while R2 from shape-and-size-based features and R3-5 from the Gray-level co-occurrence matrix. All features were standardized by the z-score method (mean and standard deviation of R1: -0.0891 and 0.239, R2: 1.60 and 0.832, R3: 0.0756 and 0.0267, R4: 0.514 and 0.0455, R5: 738 and 40.5).

Let:

***X*** denote the three dimensional image matrix with *N* voxels and **P** the first order histogram with $N_{g}$ discrete intensity levels,

$P_{C}\left( i,j \right)$ be the GLCM for distance $\delta=1$ and direction $\alpha$ (0^o^, 45^o^, 90^o^, 135^o^),

$p_{x}\left( i \right)=\sum_{j=1}^{N_{g}} P_{C}\left( i,j \right)$ be the marginal row probabilities,

$p_{y}\left( j \right)=\sum_{i=1}^{N_{g}} P_{C}\left( i,j \right)$ be the marginal column probabilities,

$HXY=-\sum_{i}^{N_{g}} \sum_{j}^{N_{g}} P_{C}\left( i,j \right)\log_{2}[P_{C}\left( i,j \right)]$ be the entropy of $P_{C}\left( i,j \right)$,

$$HXY2=-\sum_{i}^{N_{g}} \sum_{j}^{N_{g}} p_{x}\left( i \right)p_{y}\left( j \right){log}_{2}({p_{x}\left( i \right)p}_{y}\left( j \right))$$

$p_{x+y}\left( k \right)=\sum_{i}^{N_{g}} \sum_{j}^{N_{g}} P_{C}(i,j), i=j=k, k=2,3, \ldots, 2N_{g}$,

$p(i,j|\theta)$ be the $\left( i,j \right)$th entry in the given GLRLM $p$ for a direction $\theta$ (0^o^, 45^o^, 90^o^, 135^o^).

**R1 (skewness):**

$$R1=\frac{\frac{1}{N}\sum_{i=1}^{N} {(\boldsymbol{X}\left( i \right)-\bar{X})}^{3}}{{(\sqrt{\frac{1}{N}\sum_{i=1}^{N} {(\boldsymbol{X}\left( i \right)-\bar{X})}^{2}})}^{3}}$$

Where $\bar{X}$ is the mean of ***X***.

**R2 (compactness 2):**

$R2=36\pi\frac{V^{2}}{A^{3}}$.

where V denote the volume and A the surface area of the volume of interest.

**R3 (maximum probability):**

$$R3=max\{P_{C}\left( i,j \right)\}$$

**R4 (homogeneity 2):**

$$R4=\sum_{i=1}^{N_{g}} \sum_{j=1}^{N_{g}} \frac{P_{c}(i,j)}{1+{|i-j|}^{2}}$$

**R5 (sum variance):**

$$R5=\sum_{i=2}^{2N_{g}} \left( i-SE \right)^{2}p_{x+y}(i)$$

where $SE=-\sum_{i=2}^{2N_{g}} p_{x+y}\left( i \right)\log_{2}[p_{x+y}\left( i \right)]$.

# Appendix A8. Supplementary tables and figures

703 patients with PTC were collected from January, 2016 to November, 2017 according with inclusion criteria

624 patients included for segmentation and analysis

8 patients excluded with artifacts

56 patients excluded with small lesions whose visible area <5 mm in maximum diameter

15 patients excluded with the lesions impossible to be distinguished from other diseases

F**igure S1.** Recruitment pathway for patients in this study


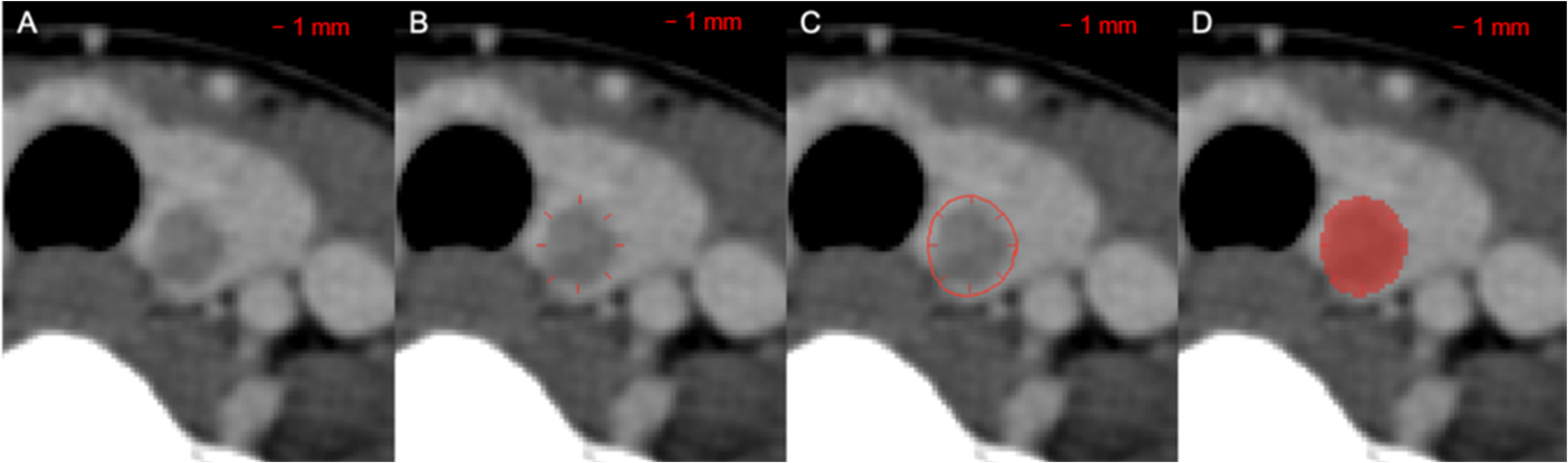


F**igure S2.** Segmentation of tumor. Figure A: Find the tumor. Figure B: Remark several surrounding points at 1 mm outer from the tumor boundary. Figure C: Draw ROI based on these points. Figure D: Complete the segmentation.


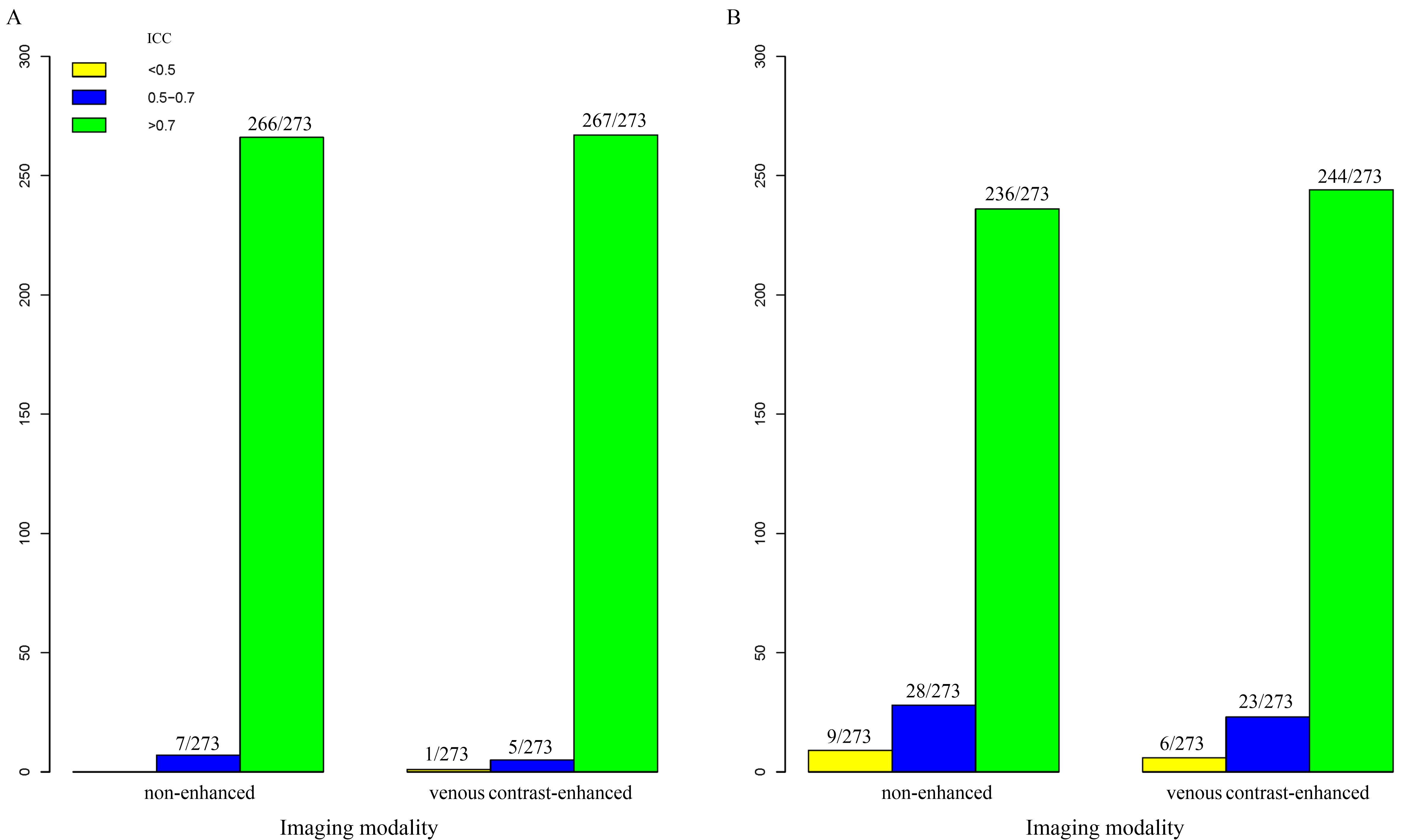


**Figure S3.** Reproducibility of extracted features. Figure A showed intra-observer agreement of the radiomic features while figure B showed inter-observer agreement of the radiomic features. Those features whose CCC is greater than 0.7 are regarded as a mark of excellent reliability and 480 (88%) features reach the criteria.

Abbreviation: CCC, class correlation coefficient.


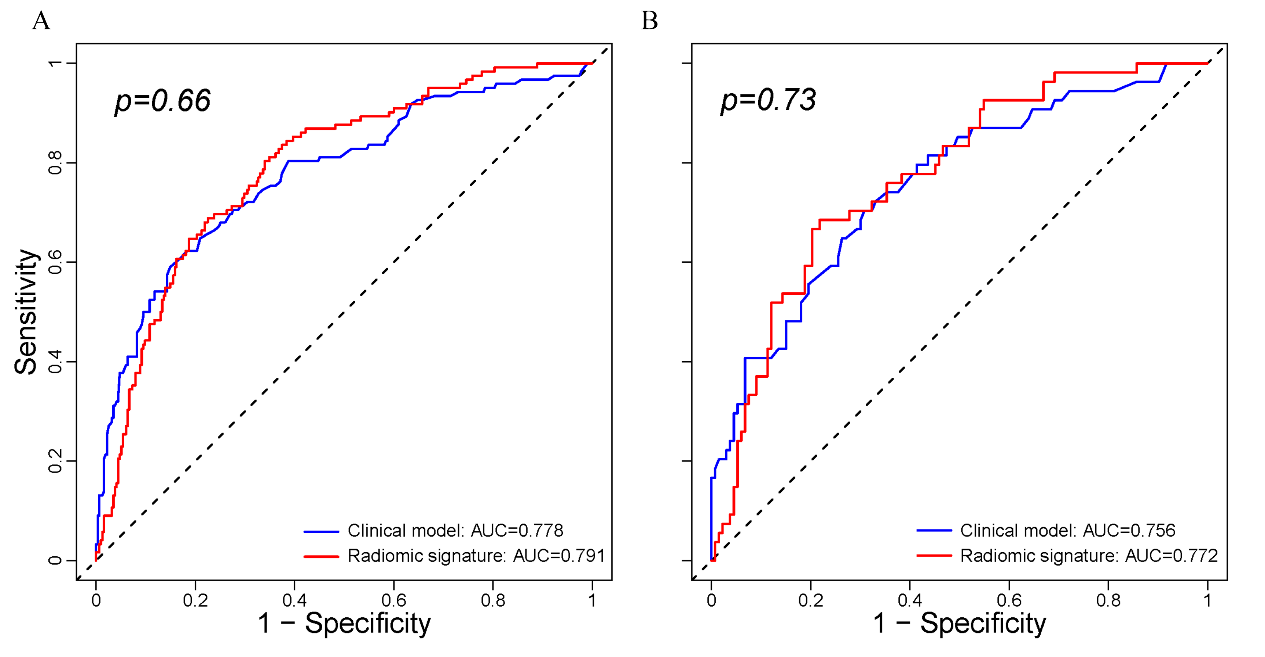


**Figure S4.** Comparison ROC curves between the clinical model and radiomic signature in training and validation cohorts.

Table S1. Characteristics of patients with PTC in the training and validation cohorts.

| Characteristics | Training Cohort (N=437) | Validation Cohort (N=187) | *p* |
| --- | --- | --- | --- |
|  |  |  |  |
| Age, mean ± SD, years | 45.04 ± 12.95 | 44.09 ± 13.64 | 0.410 |
| < 55, No. (%) | 332 (75.97) | 141 (75.40) | 0.96 |
| ≥ 55, No. (%) | 105 (24.03) | 46 (24.60) |  |
| Gender, No. (%) |  |  |  |
| Male | 80 (18.31) | 43 (22.99) | 0.215 |
| Female | 357 (81.69) | 144 (77.01) |  |
| Primary site (Location), No. (%) |  |  |  |
| Right/Left lobe | 419 (95.88) | 178 (95.19) | 0.861 |
| Isthmus | 18(4.12) | 9 (4.81) |  |
| Primary site (Position, A-P), No. (%) | |  |  |
| Ventral | 179 (40.96) | 82 (43.85) | 0.103 |
| Medium | 15 (3.43) | 1 (0.53) |  |
| Dorsal | 243 (55.61) | 104 (55.61) |  |
| Diameter, mean ± SD, mm | 12.09 ± 6.68 | 12.92 ± 7.35 | 0.168 |
| Calcification, No. (%) |  |  |  |
| Negative | 270 (61.78) | 111 (59.36) | 0.631 |
| Positive | 167 (38.22) | 76 (40.64) |  |
| BMI, mean ± SD, kg/m^2^ | 22.86 ± 3.19 | 22.72 ± 3.04 | 0.606 |
| < 25, No. (%) | 331 (75.74) | 145 (77.54) | 0.704 |
| ≥ 25, No. (%) | 106 (24.26) | 42 (22.46) |  |
| CT prediction, No. (%) |  |  |  |
| Negative | 342 (78.26) | 144 (77.01) | 0.810 |
| Positive | 95 (21.74) | 43 (22.99) |  |
| ETE, No. (%) |  |  |  |
| Negative | 315 (72.08) | 133 (71.12) | 0.883 |
| Positive | 122 (27.92) | 54 (28.88) |  |
| LN metastasis, No. (%) |  |  |  |
| Negative | 196 (44.85) | 82 (43.85) | 0.887 |
| Positive | 241 (55.15) | 105 (56.15) |  |

Table S2. Results of different combinations of feature selection methods and machine learning classifiers

| Classifiers | Feature selection methods | | |
| --- | --- | --- | --- |
|  | LASSO | PCA | mRMR |
| Logistic regression | **0.781** | 0.761 | 0.775 |
| Random forest | 0.741 | 0.698 | 0.733 |
| SVM (RBF) | 0.752 | 0.753 | 0.751 |

Abbreviation: LASSO, Least Absolute Shrinkage and Selection Operator; PCA, Principal Component Analysis; mRMR, Minimum Redundancy Maximum Relevance; SVM, Support Vector Machine; RBF, Radial Basis Function.

Table S3. Outcome measurements of models

| Models |  | Training cohort | | |  |  | Validation cohort | | |
| --- | --- | --- | --- | --- | --- | --- | --- | --- | --- |
|  | Sensitivity | Specificity | F_1_ | AUC |  | Sensitivity | Specificity | F_1_ | AUC |
| Radiomic signature | 0.689 | 0.774 | 0.729 | 0.791 |  | 0.685 | 0.737 | 0.710 | 0.772 |
| Clinical model | 0.705 | 0.724 | 0.714 | 0.778 |  | 0.685 | 0.699 | 0.692 | 0.756 |
| Radiomic nomogram | 0.770 | 0.762 | 0.766 | 0.837 |  | 0.759 | 0.707 | 0.732 | 0.812 |

Abbreviation: AUC, area under receiver operating characteristic curve.
